# Virulence-related comparative transcriptomics of infectious and non-infectious chlamydial particles

**DOI:** 10.1186/s12864-018-4961-x

**Published:** 2018-08-02

**Authors:** Thomas Beder, Hans Peter Saluz

**Affiliations:** 1 Department of Cell and Molecular Biology, Leibniz Institute for Natural Product Research and Infection Biology – Hans Knöll Institute (HKI), Beutenbergstraße 11A, 07745 Jena, Germany; 2 Network Modelling, Leibniz Institute for Natural Product Research and Infection Biology - Hans Knöll Institute (HKI), Beutenbergstraße 11A, 07745 Jena, Germany; 3 Center for Sepsis Control and Care (CSCC), Jena University Hospital, Jena, Germany; 4 Friedrich Schiller University, Fürstengraben 1, 07743 Jena, Germany

**Keywords:** Chlamydia, Comparative transcriptomics, Core transcriptome, RNA-Seq, Differential gene expression, Interspecies

## Abstract

**Background:**

Members of the phylum Chlamydiae are obligate intracellular pathogens of humans and animals and have a serious impact on host health. They comprise several zoonotic species with varying disease outcomes and prevalence. To investigate differences in virulence, we focused on *Chlamydia psittaci*, *C*. *abortus* and *Waddlia chondrophila.* Most threatening is *C*. *psittaci*, which frequently infects humans and causes psittacosis associated with severe pneumonia. The closest relative of *C*. *psittaci* is *C. abortus*, which shares the vast majority of genes but less frequently infects humans, and causes stillbirth and sepsis. *W*. *chondrophila* is more distantly related, and occasional human infections are associated with respiratory diseases or miscarriage. One possible explanation for differences in virulence originate from species-specific genes as well as differentially expressed homologous virulence factors.

**Results:**

RNA-sequencing (RNA-Seq) was applied to purified infectious elementary bodies (EBs) and non-infectious reticulate bodies (RBs) in order to elucidate the transcriptome of the infectious and replicative chlamydial states. The results showed that approximately half of all genes were differentially expressed. For a descriptive comparison, genes were categorised according to their function in the RAST database. This list was extended by the inclusion of inclusion membrane proteins, outer membrane proteins, polymorphic membrane proteins and type III secretion system effectors. In addition, the expression of fifty-six known and a variety of predicted virulence and immunogenic factors with homologs in *C*. *psittaci*, *C*. *abortus* and *W*. *chondrophila* was analysed. To confirm the RNA-Seq results, the expression of nine factors was validated using real-time quantitative polymerase chain reaction (RT-qPCR). Comparison of RNA-Seq and RT-qPCR results showed a high mean Pearson correlation coefficient of 0.95.

**Conclusions:**

It was shown that both the replicative and infectious chlamydial state contained distinctive transcriptomes and the cellular processes emphasised in EBs and RBs differed substantially based on the chlamydial species. In addition, the very first interspecies transcriptome comparison is presented here, and the considerable differences in expression of homologous virulence factors might contribute to the differing infection rates and disease outcomes of the pathogens. The RNA-Seq results were confirmed by RT-qPCR and demonstrate the feasibility of interspecies transcriptome comparisons in chlamydia.

**Electronic supplementary material:**

The online version of this article (10.1186/s12864-018-4961-x) contains supplementary material, which is available to authorized users.

## Background

Chlamydiae are obligate intracellular gram-negative bacteria that infect a wide range of hosts [1]. Within the phylum, the family *Chlamydiaceae* is most prominent and comprises the important human pathogens *Chlamydia* (*C.*) *trachomatis* and *C*. *pneumoniae* [2]. Besides *Chlamydiaceae*, there are other chlamydia-like families, such as *Waddliaceae*, that also infect humans [1, 3, 4]. All members of *Chlamydiaceae* have a very reduced genome of about 1.1 megabase pairs (Mbp) and share the majority of protein-coding genes [5]⁠. Although the genomes are highly similar, host specificity and virulence differ substantially [1, 6]. Striking examples of this are seen for the close relatives *C*. *psittaci* and *C. abortus* [7, 8]. *C. psittaci* is the causative agent of ornithosis (also known as psittacosis), the most widespread zoonotic chlamydiosis [7, 9, 10]. In birds, the symptoms include lethargy, hyperthermia, abnormal excretions and respiratory distress [11]. The sequelae of psittacosis in humans range from clinically silent or mildly flu-like to an acute illness with severe pneumonia and death, and even human-to-human transmissions of *C*. *psittaci* were reported [12–14]. *C. abortus*, the closest relative of *C*. *psittaci*, is less widespread but still of economic importance because it is the aetiological agent of abortion in sheep and goats [8] and it is able to colonise the human placenta [15]; the major threat concerns pregnant women with close contact to stillborn ruminants. The resulting infections can lead to preterm stillbirth and a sepsis-like disease [16]. *C*. *psittaci* and *C. abortus* share the majority of genes, but there are genetic differences that might be responsible for the host specificity and tissue tropism. These factors include the number of polymorphic membrane proteins (Pmps), genes within the plasticity zone (PZ) and the existence of a plasmid [5]. Other chlamydia-like organisms like *Waddlia (W.) chondrophila* also exhibit the characteristic biphasic developmental cycle and occasionally infect humans [1, 4, 17]. Additional similarities between *W*. *chondrophila* and *Chlamydiaceae* concern the functional type III secretion system (T3SS) and the presence of various homologous virulence factors [18]. However, the genome architecture of *W*. *chondrophila* is different, with a genome size of about 2.1 Mbp and double the number of genes as the *Chlamydiaceae* [18]. Furthermore, *W*. *chondrophila* encodes some virulence-related genes that are not present in *Chlamydiaceae*, but the Pmps and PZ are absent [18, 19]. The most striking similarities of all Chlamydiae is the biphasic developmental cycle and the establishment of an intracellular inclusion in which the chlamydiae reside, a structure highly modulated by the pathogen [20]. The developmental cycle is characterised by the alternation of infectious elementary bodies (EBs) and non-infectious reticulate bodies (RBs) [1, 21]. EBs are small, extracellular particles that are responsible for the dissemination and invasion of susceptible cells. After internalisation, EBs differentiate into the larger, replicative RBs. RBs exist, except for extruded inclusions, inside the host cell and after various cycles of replication they differentiate into EBs, which are released by host cell lysis [21].

In this study, we applied RNA-Sequencing (RNA-Seq) to purified EBs and RBs of *C*. *psittaci*, *C*. *abortus* and *W*. *chondrophila*. Thereby, we elucidated the transcriptomes of the infectious and non-infectious states and compared the expression of virulence and immunogenic factors. The rationale for this approach is that *C*. *psittaci* and *C. abortus* have highly similar genomes and a well-documented zoonotic potential but differ substantially in disease outcome and host preference [1, 5, 15, 22]. It is reasonable that these differences originate from the presence of species-specific genes but also due to the differential expression of homologous virulence factors. The more distant relative *W*. *chondrophila* was chosen as a third model because while infections occur in humans they are less frequent compared to *C*. *psittaci* and *C. abortus*. Moreover, *W*. *chondrophila* may infect the human respiratory tract and is also associated with miscarriage, features that resemble tissue tropism and disease outcomes of *C. psittaci* and *C. abortus* [17, 23].

## Results

### Genome comparison

The genome architecture of *C*. *psittaci* or *C. abortus* is very different when compared to *W*. *chondrophila*, with striking variations including genome size, number of genes and presence of PZ and Pmp genes (Table 1). However, *W*. *chondrophila* contains a family of unique outer membrane proteins (OMPs) [19]. Furthermore, *C*. *psittaci* and *W*. *chondrophila* WSU 86–1044 harbor a plasmid, which originated from a common ancestor [18], that is absent in *C. abortus* (Table 1).

**Table 1:** General genomic features of *C*. *psittaci*, *C*. *abortus* and *W*. *chondrophila*

Feature	*C*. *psittaci* 02DC15^a^	*C. abortus* S26/3^b^	*W. chondrophila* WSU 86-1044^f^
Chromosome size (nt)	1,172,182	1,144,377	2,116,312
Contigs	2^b^	1	2
Plasmid size (nt)	7557^b^	–	15,593
G + C content (%)	39	40	44
Genes	1023	1009	1919
Protein-coding genes	975	933	1863
Pmps	22^a + c^	16^d + e^	–
Protein-coding genes in the PZ	16	6	–
Pseudo genes	6	34	12
tRNA	38	38	37
rRNA operons	1	1	1

^a^ https://www.ncbi.nlm.nih.gov/genome/839?genome_assembly_id=169284

^b^This study

^c^Voigt et al. (2012)

^d^ https://www.ncbi.nlm.nih.gov/genome/1091?genome_assembly_id=300526

^e^Thomson et al. (2005)

^f^ https://www.ncbi.nlm.nih.gov/genome/?term=Waddlia+chondrophila

**Fig. 1:**
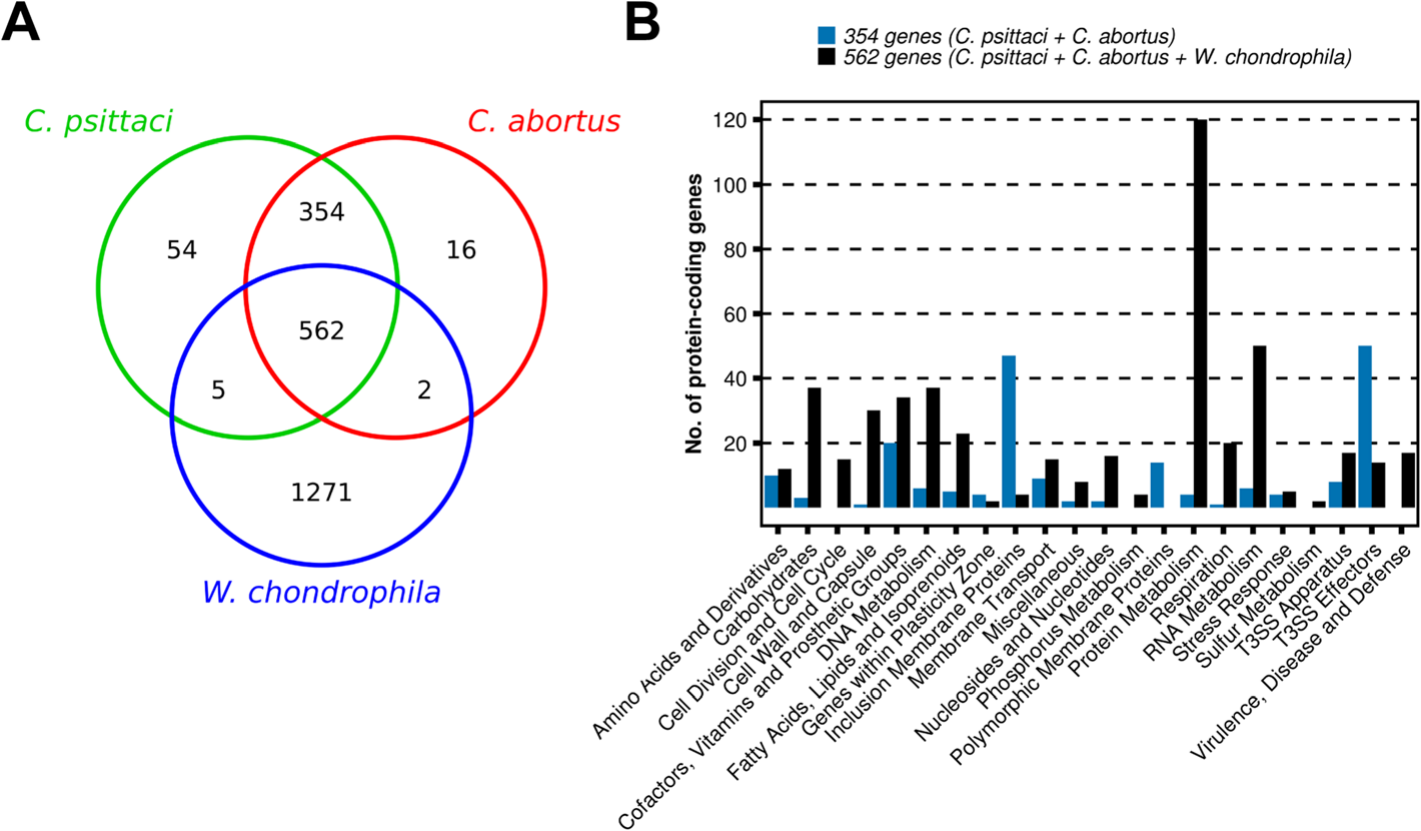
Annotation of homologous protein-coding genes in *C. psittaci*, *C. abortus* and *W. chondrophila*. (**a**) Venn diagram showing the number of protein-coding genes that are unique or shared among *C. psittaci*, *C. abortus* and *W. chondrophila.* (**b**) The 562 homologous genes in all three pathogens are mainly involved in fundamental cellular processes, whereas the 354 genes shared only among *C. psittaci* and *C. abortus* encode mainly virulence-related genes like inclusion membrane proteins, polymorphic membrane proteins and type three secretion system effectors

The number of species-specific and homologous genes in *C*. *psittaci*, *C*. *abortus* and *W*. *chondrophila* are shown in Fig. 1a. Altogether 562 homologs present in all three pathogens were identified. The genes participate in essential cellular processes like respiration, cell division, DNA, fatty acid, protein and RNA metabolism (Fig. 1b). Functional categories of the genes were assigned to the RAST database. To extend the list of categories, T3SS effectors were predicted with a previously published SVM approach and EffectiveT3. Further, predictions of inclusion membrane proteins (Incs), OMPs and Pmps were assembled from the literature as described in the Methods. However, potential virulence-related genes among the homologs from all three pathogens were also found, mainly involved in cell wall synthesis, membrane transport and formation of chlamydial OMC. The close relatives to *C*. *psittaci* and *C. abortus* share the majority of 916 protein-coding genes, of which 354 were not present in *W*. *chondrophila* (Fig. 1a). The genes mainly encode Pmps, Incs and T3SS effectors (Fig. 1b), all of which are classes of proteins involved in virulence [24]. Beside these homologs, there are few species-specific genes, i.e., fifty-four in *C*. *psittaci* and only sixteen in *C. abortus* (Fig. 1a).

### Raw data processing and coverages

A summary of the trimming, assembly and alignment results is shown in Additional file 1: Figure S1, left panels). High sequencing depths were achieved for all three organisms in the range of 47 to 603 genome coverages (Additional file 2). Distribution of aligned reads to various genome locations are also shown in Additional file 1: Figure S1, right panels). Most reads mapped to mRNAs and thus resulted in high sensitivity: 96% in *W*. *chondrophila* and in *C. psittaci* and *C. abortus* virtually all protein-coding genes were ascertained to be expressed.

### Intraspecies comparison of gene expression in EBs and RBs

**Fig. 2:**
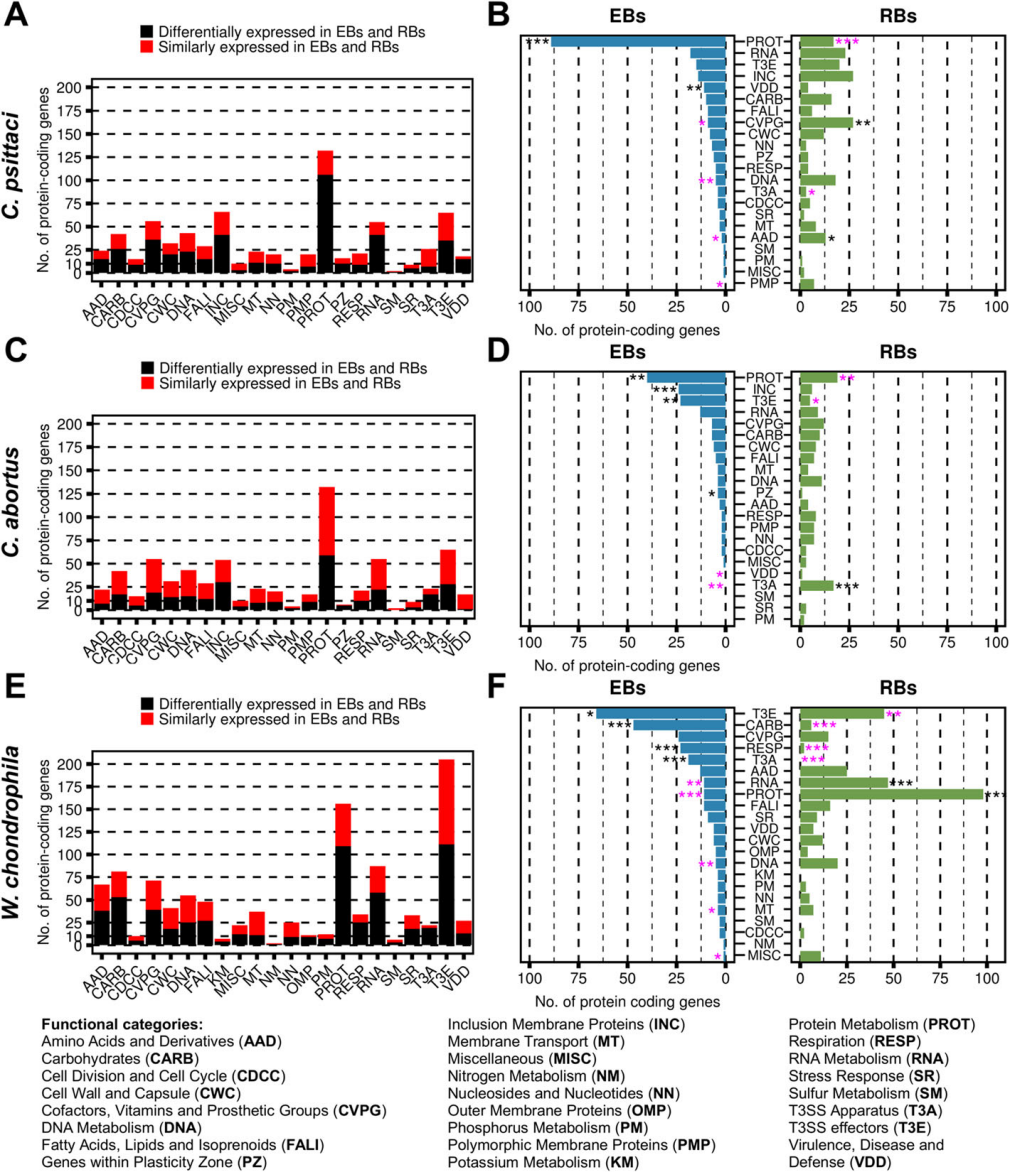
EBs and RBs show highly diverse gene expression in *C. psittaci*, *C. abortus* and *W. chondrophila*. The left panel shows the number of similarly and differentially expressed genes in the assigned functional categories for *C. psittaci* (**a**), *C. abortus* (**c**) and *W. chondrophila* (**e**). About half of all genes are differentially expressed in EBs and RBs (black) in all three species. The right panel shows the number of up-regulated genes in EBs (blue) and RBs (green) in *C*. *psittaci* (**b**), *C*. *abortus* (**d**) and *W*. *chondrophila* (**f**) within each functional category. Overrepresentation (black asterisks) and underrepresentation (magenta asterisks) of genes within the functional categories are indicated. Significance threshold is depicted by asterisks and represent *p*-value < 0.05 (*), *p*-value < 0.01 (**) and *p*-value < 0.001 (***)

The raw counts, normalised expression levels in EBs and RBs and functions of the corresponding proteins are listed in Additional files 3, 4 and 5. In *C*. *psittaci*, 601 genes (61.6%) were differentially expressed (Fig. 2a), whereby 290 were upregulated in EBs and 311 in RBs. An enrichment analysis of categorised genes was performed in order to transfer biological functions to the high number of differentially expressed genes. The most prominent biological category in *C. psittaci* EBs was “Protein Metabolism”, in which the expression of eighty-nine genes was upregulated (Fig. 2b). A second category was overrepresented in EBs, i.e., “Virulence, Disease and Defense”, which comprised virulence factors (see Additional file 3). Several categories, however, were underrepresented in EBs: “Amino Acids and Derivatives”, “Cofactors, Vitamins and Prosthetic Groups”, “DNA Metabolism” and “Polymorphic Membrane Proteins”. In RB transcripts, the categories “Amino Acids and Derivatives” and “Cofactors, Vitamins and Prosthetic Groups” were overrepresented, whereas transcripts in the category “Protein Metabolism” were underrepresented (Fig. 2b). Due to the genomic similarities of *C*. *psittaci* and *C. abortus*, species-specific genes might have great impact on the increased virulence of *C*. *psittaci*. A known virulence factor present in *C*. *psittaci* but not in *C. abortus* or *W*. *chondrophila* is the MACPF domain gene (CPS0B_RS02865) located in the PZ that was similarly expressed in EBs and RBs (Additional file 6).

Virtually all *C*. *psittaci* isolates contained a 7.5 kb plasmid that was absent in *C. abortus*. The plasmid in chlamydia is known to be involved in infectivity and virulence [25, 26] and in *C*. *psittaci* it contains eight genes, seven of which are encoded on the plus strand. Plasmid reads represented 1.49% of the RB and 0.67% of the EB libraries, respectively, and three genes, *pgp1*, *pgp2* and *pgp8*, were differentially expressed in EBs and RBs (Additional file 6). Of these, the DNA helicases *pgp1* and *pgp2* were more abundant in EBs, whereas the integrase *pgp8* was upregulated in RBs.

In *C. abortus*, 397 genes (42.6%) were differentially expressed (Fig. 2c), 207 of which were upregulated in EBs and 190 in RBs, respectively (Additional file 4). Like in *C*. *psittaci*, most of the genes upregulated in EBs belonged to the category “Protein Metabolism” (Fig. 2d). Other categories that were overrepresented in *C. abortus* EBs were “Predicted Incs”, “T3SS effectors” and “Plasticity Zone”. In *C. abortus* RBs, there was only one overrepresented category, “T3SS Apparatus” (Fig. 2d). Among the sixteen *C. abortus*-specific genes (Fig. 1a), no known virulence factor was found, but a comprehensive description of these genes and their potential functions are discussed in the Additional file 7.

In *W*. *chondrophila*, 998 genes (54.2%) were differentially expressed (Fig. 2e), of which 479 were upregulated in EBs and 519 in RBs (Additional file 4). Again, the category “Protein Metabolism” contained most differentially expressed genes, but in contrast to *C*. *psittaci* and *C. abortus*, this category was overrepresented in RBs (Fig. 2f). A second overrepresented category in RBs was “RNA Metabolism”, whereas in EBs “T3SS Effectors”, “T3SS Apparatus”, “Carbohydrate Metabolism” and “Respiration” were overrepresented (Fig. 2f).

From the 1271 *W*. *chondrophila* specific genes (Fig. 1a), most are involved in metabolic processes but some encode known virulence factors that participate in membrane transport, drug resistance or belong to the *W*. *chondrophila ompA* family; several were differentially expressed in EBs and RBs (see Additional files 6 and 7 for discussion).

### Expression of homologous genes in *C*. *psittaci* and *C. abortus*

**Fig. 3:**
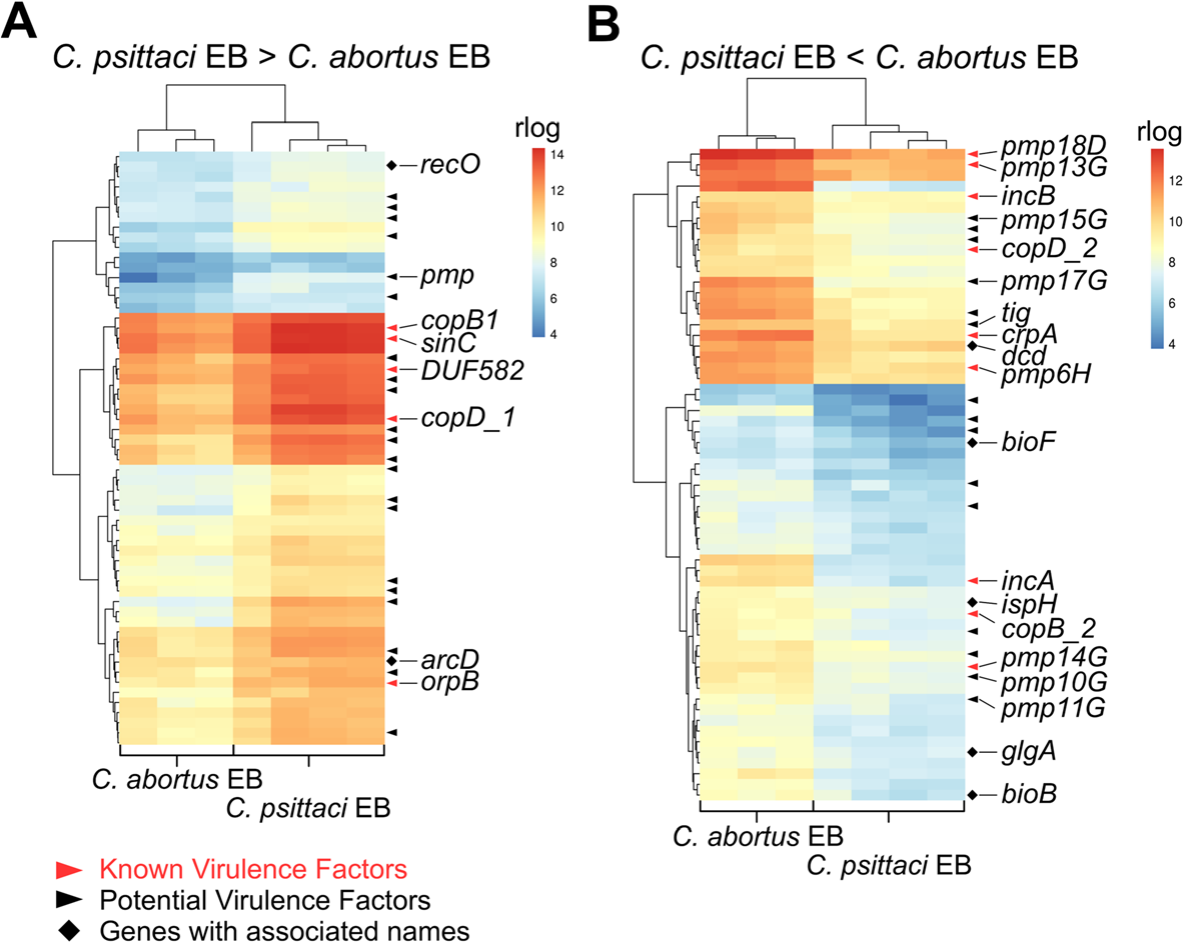
Heatmaps showing differentially expressed transcripts in EBs of *C. psittaci* and *C. abortus*. From the 354 homologous genes shared between *C. psittaci* and *C. abortus* 120 were differentially expressed (Benjamini-Hochberg adjusted *p*-value < 0.01 and an absolute log2-fold change > 1.0) in EBs. Of these 59 are up-regulated in *C. psittaci* (**a**) and 61 in *C. abortus* (**b**). Known as well as potential virulence factors (predicted Incs and T3SS effectors) and genes with associated gene names are indicated. Hierarchical clustering was performed using hclust with regularized log transformed (rlog) gene expression values and the complete linkage method

Differential expression of homologous genes (especially virulence factors) might be of great relevance in the chlamydia disease outcome. The expression of 354 homologous genes shared between *C*. *psittaci* and *C. abortus* (Fig. 1a) is shown in Additional file 8. In EBs, 120 (34%) of these 354 genes were differentially expressed, whereas in *C*. *psittaci* EBs, fifty-nine transcripts were more abundant than in *C. abortus* (Fig. 3a). Among these are five known and twenty-one putative virulence factors like Incs, Pmps and T3SS effectors. Four known and six putative virulence factors, along with five genes that encode hypothetical proteins, formed a cluster of highly expressed genes (Fig. 3a). In *C. abortus* EBs, sixty-one transcripts were more abundant than in *C*. *psittaci* (Fig. 3b); these transcripts comprised nine known and twelve putative virulence factors (particularly Pmps).

**Fig. 4:**
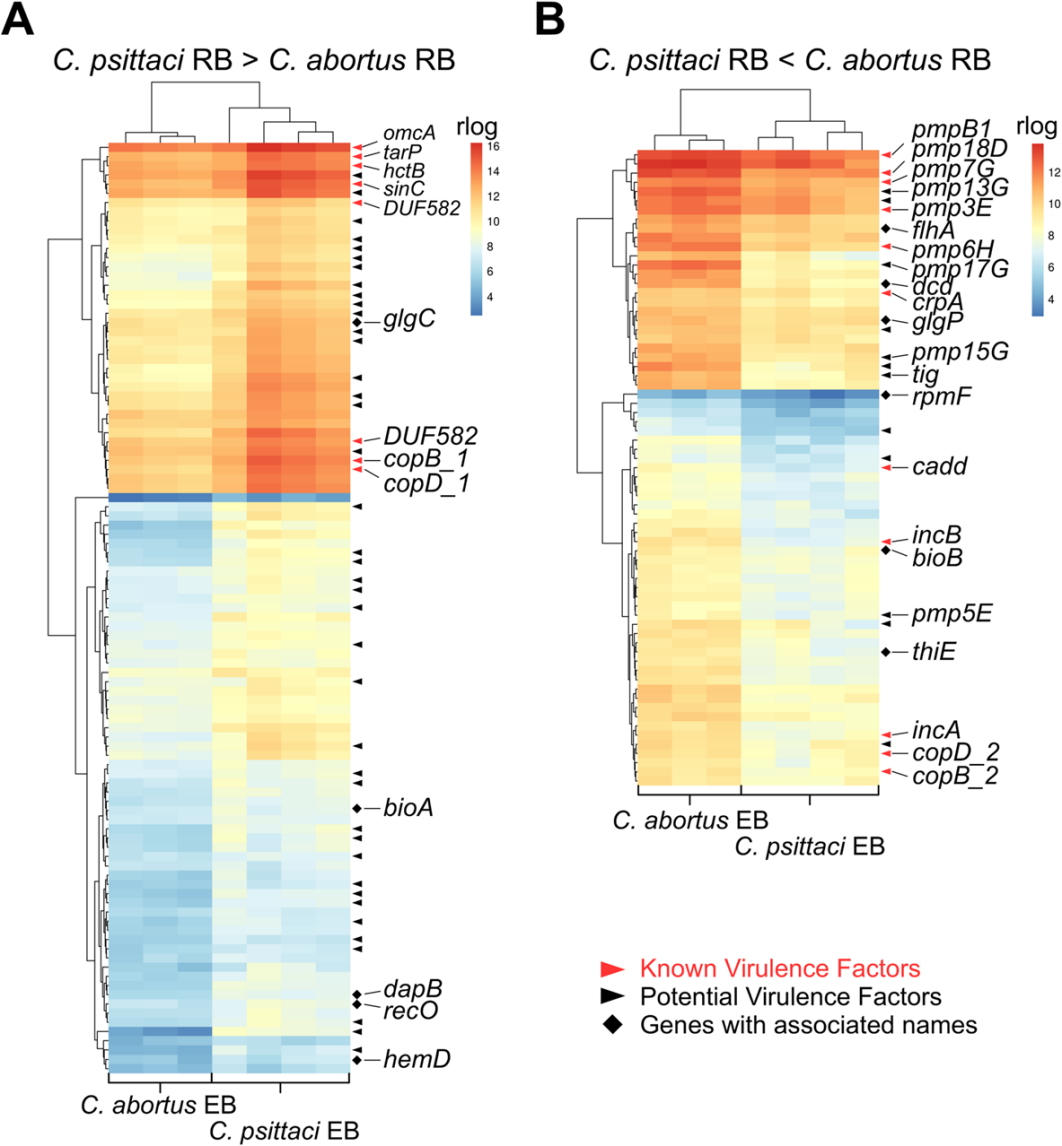
Heatmaps showing differentially expressed transcripts in RBs of *C. psittaci* and *C. abortus*. From the 354 homologous genes shared between *C. psittaci* and *C. abortus* 170 were differentially expressed (Benjamini-Hochberg adjusted *p*-value < 0.01 and an absolute log2-fold change > 1.0) in RBs. Of these 101 are up-regulated in *C. psittaci* (**a**) and 69 in *C. abortus* (**b**). Known as well as potential virulence factors (predicted Incs and T3SS effectors) and genes with associated gene names are indicated. Hierarchical clustering was performed using hclust with regularized log transformed (rlog) gene expression values and the complete linkage method

In RBs, 170 (48%) genes were differentially expressed. The 101 transcripts more abundant in *C*. *psittaci* RBs are shown in Fig. 4a and included eight known virulence factors. In addition, the transcripts of forty potential virulence factors, of which most are predicted T3SS effectors, were more abundant in RBs of *C*. *psittaci* than *C. abortus*. Noticeably, there was a cluster of highly expressed genes comprised of four known virulence factors (*hctB*, *omcA*, *sinC* and *tarP*) and two predicted T3SS effectors.

In *C. abortus* RBs, sixty-nine transcripts were more abundant than in *C*. *psittaci*. These transcripts included eleven known (like the Pmps that form a cluster of highly expressed genes) and twelve putative virulence factors (Fig. 4b). More detailed heatmaps of differentially expressed genes in *C*. *psittaci* and *C. abortus* that include NCBI locus tags and annotation of genes are shown in Additional file 1: Figures S2 – S5).

**Fig. 5:**
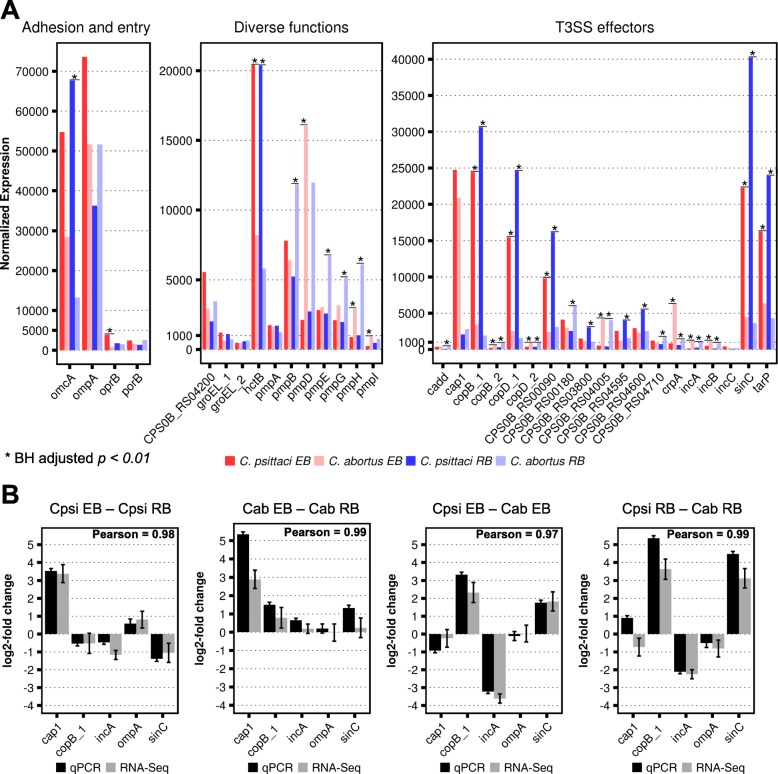
Expression of homologous genes shared between *C. psittaci* and *C. abortus*. **a** The normalized expression (DESeq2) of 34 virulence and immunogenic factors present in *C. psittaci* and *C. abortus* but absent in *W. chondrophila* are shown. Significance threshold is depicted by asterisk (*) and represents Benjamini-Hochberg adjusted *p*-value < 0.01 and an absolute log2-fold change > 1.0. Homologous virulence factors for which no gene name was found are specified with the *C. psittaci* NCBI locus tag. **b** Confirmation of the RNA-seq results with RT-qPCR for five genes shared among *C. psittaci* and *C. abortus*. The Pearson correlation coefficient (> 0.97) demonstrates the high degree of correlation between the RT-qPCR and RNA-seq results in both the intra- and inter-species comparison of gene expression. Standard deviation of log2-fold changes are indicated

The expression of thirty-four known virulence factors and immunogenic genes shared between *C*. *psittaci* and *C. abortus* is summarised in Fig. 5a. Some virulence factors, like *ompA*, *porB*, *pmpA* and *cap1*, were similarly expressed in the EBs and RBs of both organisms; *ompA* was one of the most abundant transcripts. *sinC* and *tarP* transcripts were more highly expressed in EBs and RBs of *C*. *psittaci* compared to *C. abortus*. In contrast, *pmpD*, *pmpH*, *incA* and *incB* were more highly expressed in *C. abortus*. To confirm these results, we used RT-qPCR to evaluate the expression of *cap1*, *copB_1*, *incA*, *ompA* and *sinC*. We found a high level of correlation (> 0.97) between RT-qPCR and RNA-Seq results in this interspecies comparison (Fig. 5b).

### Expression of homologous genes in *C*. *psittaci*, *C*. *abortus* and *W*. *chondrophila*

*C*. *psittaci*, *C*. *abortus* and *W*. *chondrophila* shared 562 homologous genes (Fig. 1a) and the functions and expression of these are listed in Additional file 9. These genes also comprise homologs of general stress response proteins (e.g., DnaK, GroEL, and GroES) that are involved in development (e.g., HctA), adhesion (e.g., OmcB) and drug-resistance (e.g., PhnP).

In *C*. *psittaci* and *C. abortus* EBs, *C. psittaci* and *C. abortus* 444 (79%) genes were similarly expressed, whereas *C*. *psittaci* and *W*. *chondrophila* expressed 319 (57%) and *C*. *abortus* and *W*. *chondrophila* 310 genes (55%) similarly. These results demonstrate that in *C*. *psittaci* and *C. abortus* EBs, *C. psittaci* and *C. abortus*expression of core genes is more similar than when compared to *W*. *chondrophila*. In addition, there was a core transcriptome in EBs that consisted of 209 (37%) genes that were similarly expressed in all three pathogens; it comprised *hrta*, *mgtE*, *phnP* and *sodA* (Additional file 9).

In RBs, there were only 327 (58%) genes similarly expressed in *C*. *psittaci* and *C. abortus*. When compared to RBs of *W. chondrophila*, *C. psittaci* expressed 304 genes (54%) and *C. abortus* expressed 345 (61%) similarly. There was also a core transcriptome in RBs that consisted of 177 (31%) similarly expressed genes in all three pathogens, including *cpaF*, *dnaK*, *gp6D*, *hctA*, *hrtA*, *marC*, *mgtE* and *zntA* (Additional file 9).

**Fig. 6:**
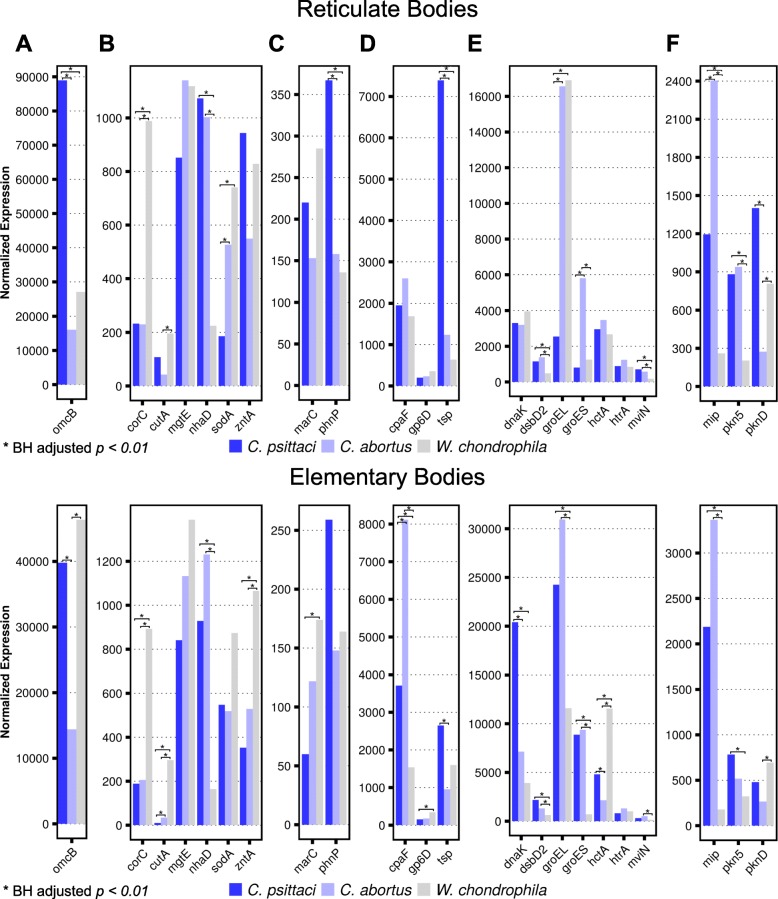
Expression of homologous genes shared between *C. psittaci*, *C. abortus* and *W. chondrophila*. The inter-species comparison revealed that various genes are differentially expressed in EBs and RBs. The normalized expression (DESeq2) of 22 genes (mostly virulence factors) shared among *C. psittaci*, *C. abortus* and *W. chondrophila* are shown. Genes are sorted according to their functions: **a** adhesion and host cell entry, **b** stress response, **c** drug resistance, **d** manipulation of host cell immune response, **e** diverse functions and **f** effectors secreted by the type III secretion system. Significance threshold is depicted by asterisk (*) and represents Benjamini-Hochberg adjusted *p*-value < 0.01 and an absolute log2-fold change > 1.0

The most abundant transcripts of the core genome were *omcB* and *groEL*, and the expression of these and other known genes shared among *C*. *psittaci*, *C*. *abortus* and *W*. *chondrophila* is shown in Fig. 6. The interspecies comparison resulted in several variants of expressed genes that ranged from similarly to differentially expressed genes in the infectious and non-infectious states of all three species. For example, the expression level of *htrA* was similar in EBs and RBs from all three organisms. In contrast, genes like *hctA*, *cpaF*, *dnaK*, *marC* and *zntA* were similarly expressed in RBs but differentially in EBs, and other genes (e.g., *sodA* and *phnP*) were similarly expressed in EBs from all three pathogens but differentially expressed in RBs (Fig. 6). *omcB* represented a differentially expressed virulence factor. It is an important component of the chlamydial OMC and one of the most abundant transcripts of the core genome (Additional file 9). It was highly expressed in EBs of *C*. *psittaci* and *W*. *chondrophila* but much less in *C. abortus* (Fig. 6). In order to confirm the RNA-Seq results, we performed RT-qPCRs with primers selected for *dnaK*, *hctA*, *mip* and *omcB*. We observed a high correlation between RT-qPCR and RNA-seq results (see Additional file 1: Figure S6).

## Discussion

### Comparison of gene expression in EBs and RBs

We showed that infectious and non-infectious chlamydial particles contain distinctive transcriptomes, as previously reported for *C*. *trachomatis* and *C*. *pneumoniae* [27, 28]. However, the cellular processes emphasised in EBs and RBs differ substantially depending on the chlamydial species (Fig. 2).

High expression of genes involved in “Protein Metabolism” in *C*. *psittaci* and *C. abortus* EBs might be due to newly synthesised proteins that are required upon chlamydial infection. High expression of the proteinaceous components of the 30S and 50S ribosomal subunits in early infection was also shown in *C*. *trachomatis* and *C*. *pneumoniae* EBs and in early chlamydial infection [27–30]. In addition, upregulation of Pmps, Incs and genes involved in the T3SS apparatus formation in *C. abortus* RBs is consistent with the infection model postulated by [31].

A substantial difference between *C*. *psittaci* and *C. abortus* the presence of a plasmid in *C*. *psittaci.* The plasmid of *C. trachomatis* and *C*. *muridarum* is known to be involved in the infectivity and virulence and plasmid proteins regulate the transcription of multiple chromosomal genes in these species [25, 32]. Interestingly, the important human and zoonotic pathogen, *C*. *pneumoniae*, does not normally harbor a plasmid and a study of the *C*. *psittaci* 6 BC strain showed no effect of plasmid loss in a murine model [33]. This indicates differences in the impact of the plasmid on virulence among chlamydial species, which might associated with the various niche adaptions of the pathogens.

In *W*. *chondrophila*, increased RNA and protein synthesis was indicated during replication, whereas T3SS apparatuses and effectors were synthesised in the EBs (Fig. 2). Moreover, there might be respiratory activity in *W*. *chondrophila* EBs, as previously proven for another environmental chlamydia [34], since the categories “Carbohydrates” and “Respiration” were overrepresented in the infectious state.

Some upregulated transcripts in EBs might represent “carryover mRNAs” (e.g., the histone-like genes *hctA* and *hctB*) that are important at the end of the life cycle but degraded early after infection [35]. Other mRNAs that are required in early infection might be preloaded into EBs in order to accelerate corresponding protein synthesis. An example is the virulence factor Cap1, an Inc. protein associated with capture of lipid droplets [36] and highly expressed in EBs (Fig. 5a)*.* Its high expression might correspond to the demand for lipids in order to form membranes after chlamydia internalisation.

### Expression of genes shared between *C*. *psittaci* and *C. abortus*

This study is the first reported interspecies comparison and therefore emphasis was put on the confirmation of the RNA-Seq results. For this, RT-qPCR was used to cross-validate the expression of nine homologous factors that represented low, high as well as similarly and differentially expressed genes. A high correlation between RT-qPCR and RNA-Seq was found in both the intraspecies (e.g. EBs with RBs of *C*. *psittaci*) and interspecies (e.g. EBs from *C*. *psittaci* with *C. abortus*) comparisons and demonstrated the validity of our approach (Fig. 5b; Additional file 1: Figure S6).

There were large differences in the expression of genes involved in virulence, especially in EBs, whereas most of the genes involved in metabolic processes were similarly expressed. It is reasonable that differential expression of virulence factors contributes to the differences between *C*. *psittaci* and *C. abortus*. For example, *sinC* and *tarP* were more highly expressed in *C*. *psittaci* compared to *C. abortus* (Fig. 5a). Translocated actin recruiting phosphoprotein (TARP) is translocated into the host cytosol upon EB attachment [37] and is the most abundant T3SS effector in *C*. *trachomatis* EBs [38]. Although the detailed mechanism of chlamydial entry has yet to be elucidated, evidence suggests that TARP mediates chlamydia internalisation [39]. It facilitates the recruitment of actin filaments to the site of EB attachment, and the high *tarP* expression in *C*. *psittaci* EBs might contribute to its higher infectivity by promoting EB internalisation [40]. *sinC* is conserved in *C*. *psittaci*, *C. abortus*, *C*. *caviae* and *C. felis*, but surprisingly not in the major human pathogens *C*. *trachomatis* and *C*. *pneumoniae* [41]*.* SinC is secreted and targets a conserved component of the inner nuclear membrane [41]. It apparently alters nuclear envelope functions of the infected host cell and, consequently, higher expression in *C*. *psittaci* might contribute to increased virulence when compared to *C. abortus* [41].

In contrast, *incA* and *incB* are more highly expressed in *C. abortus* EBs and RBs (Fig. 5b). Incs are located at the interphase of the inclusion and the host cytosol and thus are important virulence factors; they inhibit fusion with endosomal compartments or promote fusogenicity with host vesicles [42]. *C*. *psittaci* IncB associates with the host protein Snapin and connects chlamydial inclusions with the microtubule network [43], whereas IncA interacts with the host protein G3BP1. This action leads to a decreased concentration of the c-Myc protein [44], and upregulation of *incA* might contribute to apoptosis inhibition in *C. abortus*.

Despite these differences there were similarly expressed virulence factors, such as *ompA*, *porB* and *pmpA* and *cap1*, between *C*. *psittaci* and *C. abortus* that might contribute to the resembling features of the pathogens (Fig. 5b). OmpA, PorB and PmpA are components of the OMC, whereby OmpA is the most abundant OMC protein and is involved in protective immunity against chlamydia [45, 46]. In concordance, *ompA* was also one of the most abundant transcripts found in *C*. *psittaci* and *C. abortus* as well as in *C*. *trachomatis* and *C*. *pneumoniae* EBs [27, 28].

### Expression of homologous genes in *C*. *psittaci*, *C*. *abortus* and *W*. *chondrophila*

In order to gain more profound insights into chlamydial gene expression, *W*. *chondrophila* was included in the analysis. In total, 562 homologous genes (e.g., *hctA*, *hrtA* and *omcB*) present in all three pathogens were identified (Fig. 1a). These genes participate in essential processes like stress response, respiration, development, DNA, fatty acid, protein and RNA metabolism (Fig. 1b**)**. HctA is involved in chromosome condensation at the end of the developmental cycle [47], and the highest *hctA* expression was detected in *W*. *chondrophila* EBs, followed by *C*. *psittaci* EBs, and the lowest expression was in *C. abortus* (Fig. 6). Expression of the histone–like *hctA* might directly influence the duration of the pathogen life cycle. Correspondingly, *hctA* expression was in accordance with the life cycle of each microorganism in human cells: *W*. *chondrophila* requires 30 h, followed by *C*. *psittaci* (38 h) and *C. abortus* (48 h).

*C. trachomatis* HrtA is a temperature-activated serine protease specific for unfolded proteins [48] and has important functions in stress resistance [49]. OmcB mediates initial contact with the host cell [50] and the transcript is enriched in *C. trachomatis* EBs [27]. High *omcB* expression in *C*. *psittaci* and *C*. *trachomatis* EBs might directly influence attachment to the host cell and, consequently, cause increased infectivity of the pathogens when compared to *C. abortus* or *W*. *chondrophila*.

## Conclusion

We showed that both the replicative and infectious chlamydial state contained distinctive transcriptomes and that the cellular processes emphasised in EBs and RBs differ substantially within the chlamydial species. Further, we present here the very first interspecies transcriptome comparison and found considerable differences in the expression of homologous virulence factors. To confirm the RNA-Seq results, the expression of nine homologous genes was cross-validated using RT-qPCR; a high correlation was found. Differential expression of homologous virulence factors might directly influence infectivity, host specificity and tissue tropism of the pathogens. It is important to note that harvesting time points have to be considered when focusing on an interspecies comparison of RB and EB transcriptomes. RBs and EBs of the three pathogens were purified at different time points in order to isolate only characteristic developmental forms, e.g., by exclusion of RBs to EBs transition states and vice versa, also referred to as intermediate bodies. Our findings may not apply to forms at other time points during infection. However, a time-resolved transcriptome analysis of RBs and EBs is now possible due to the presented approach and decreasing costs for RNA-Seq.

## Methods

### Cell line, bacterial strains and purification of EBs and RBs

HEp-2 human epithelial cells (ATCC no. CCL-23) were infected with *C*. *psittaci* 02 DC15, *C. abortus* S26/3 or *W*. *chondrophila* 2032/99. *C*. *abortus* and *C*. *psittaci* infections were carried out with a multiplicity of infection (MOI) of 5.0, and *W*. *chondrophila* infection had a MOI of 0.5. This difference was done due to the devastating effects of higher *W*. *chondrophila* MOIs on the cell monolayer. For each experiment, the infected cell monolayers from eight 25 cm^2^ cell culture flasks were pooled by scraping in ice-cold sucrose-phosphate-glutamic acid (SPG) buffer. Cultivation, harvest and purification of chlamydia were carried out as previously described [51]. In order to isolate only characteristic developmental forms (exclusion of transition states of RBs to EBs and vice versa, also referred to as intermediate bodies), the chlamydial developmental cycle was monitored using fluorescence microscopy. Phases of exponential expansion of the inclusions were determined by measuring the inclusion sizes with ImageJ. Inclusion size among the species is similar and expansion of the chlamydial inclusion is linked to bacterial replication [52–55]. Time points for RB purification were chosen during the logarithmic growth phase when the inclusions were in the range of 4–6 μm in all species. Consequently, RBs were purified at the following hours post-inoculation (hpi): *C*. *psittaci*, 24 hpi; *C. abortus*, 36 hpi; *W*. *chondrophila*, 18 hpi. Time points for EB purification were chosen just before host cell rupture and release of the particles in order to collect the EBs within the first propagation cycle for all species. Thus, EBs were purified as follows: *C*. *psittaci*, 38 hpi; *C. abortus*, 48 hpi; *W*. *chondrophila*, 30 hpi. We collected four (*C*. *psittaci*) or three (*C. abortus* and *W. chondrophila*) biological replicates of purified EBs and RBs.

### RNA isolation and complementary DNA (cDNA) library preparation

EB and RB pellets were incubated in 500 μL TRIsure™ reagent (Bioline) and lysed at 65 °C for 5 min followed by phenol-chloroform extraction. The aqueous phase was mixed with one-tenth the volume of sodium acetate (3 M; pH = 5.2) and precipitation was carried out with ethanol. In total, 10 μg of RNA per sample was treated with 10 U of DNase I (Thermo Scientific) for 45 min at 37 °C. After DNase I digestion, RNA molecules longer than 200 nucleotides were purified using the RNA Clean & Concentrator™-5 kit (ZymoResearch). The absence of DNA was ensured by RT-qPCR. To remove ribosomal RNA (rRNA), the ScriptSeq™ Complete Gold Kit (Epidemiology) was used. RNA quality controls were performed after total RNA isolation, DNA digestion and rRNA removal using Bioanalyzer 2100 measurements and the Agilent RNA 6000 Pico kit (Agilent Technologies). For the synthesis of cDNA libraries, 5 ng chemically fragmented RNA was applied as described by the ScriptSeq™ Complete Gold Kit (Illumina). The cDNA libraries were barcoded using ScriptSeq™ Index PCR Primers (Illumina) and after PCR amplification were purified using the AMPure XP System (Beckman Coulter). The size distribution of cDNA and absence of primer dimers were monitored via the Agilent High Sensitivity DNA Kit (Agilent Technologies). Final libraries were sequenced by StarSEQ GmbH (Mainz) using a NextSeq 500 (Illumina) platform and the 150 bp paired-end protocol. Raw sequence data were deposited in the NCBI Sequence Read Archive under accession numbers SRP131747, SRP131830 and SRP131936.

### Sequence analysis and statistics

Reads were trimmed using Trimmomatic [56] and quality of trimmed reads was assessed using FastQC. Because of the overall short-length distribution of cDNA, overlapping paired-end reads were merged using PEAR software [57]. The assembled 36–292 bp single-end reads were aligned to human (hg19), mitochondrial (NC_012920.1) and corresponding chlamydial (NC_017292, NC_004552 or NC_014225) reference genomes using Bowtie2 in “--very-sensitive” mode [58]. For *W*. *chondrophila* 2032/99, only a draft genome was publicly accessible and therefore read alignment was performed to the closest relative, *W*. *chondrophila* WSU 86–1044 (99% sequence identity), for which the complete genome sequence was available. Alignment to the human genome was performed to determine the number of unaligned reads and assess the integrity of the sequenced strains to the available genome sequences. Similar percentages of unaligned reads (< 5%) were found for both the “in-house” sequenced *C*. *psittaci* 02 DC15 (NC_017292) and the public *C*. *abortus* and *W*. *chondrophila* genome sequences. Previously unaligned reads for *C*. *psittaci* 02 DC15 were assembled using trinityrnaseq-2.0.4 [59] and a plasmid was found that is identical to *C*. *psittaci* 6 BC (NC_017288.1)*.* Raw count tables were generated using the GenomicAlignments v1.4.2 package, whereby chlamydial rRNA and transfer RNA (tRNA) were excluded from analyses to overcome the variable efficiency of size selection and rRNA depletion steps. Differential gene expression analysis was performed using DESeq2, which normalises sequencing depth between samples using a size factor that allows for inter-sample comparisons [60]. DESeq2 adjusted the *p*-values for multiple testing using the procedure of Benjamini and Hochberg, which tests the null hypothesis that the change between treatment and control for a gene’s expression is exactly zero. For the intraspecies comparison of gene expression in EBs and RBs, a significance threshold of the Benjamini-Hochberg adjusted *p*-value (*p*_*adj*_) < 0.05 was applied.

### RT-qPCR

For each biological replicate of *C*. *psittaci*, *C*. *abortus* and *W*. *chondrophila* EBs and RBs, 100 ng DNAse I-digested RNA was reverse transcribed using iScript™ Reverse Transcription Supermix (Bio-Rad) according to manufacturer’s protocol. For RT-qPCR, cDNA was diluted 1:5 in 10 mM Tris-HCl (pH 8.0), 0.1 mM EDTA. PCR was performed in a 10 μL volume that contained 1 μL cDNA, 5 μL SsoFast™ EvaGreen® Supermix with Low ROX (Bio-Rad) and 200 nM forward and reverse primers (Additional file 10). RT-qPCR was performed with a StepOnePlus™ (Applied Biosystems) system. All RT-qPCR reactions were performed in triplicate. The primers used in the study are listed in Additional file 9. *16S rRNA* was previously applied in a variety of studies to normalise chlamydial gene expression [35]; however, it was not among the best candidates for housekeeping genes in *C*. *psittaci*, *C*. *abortus* and *W*. *chondrophila*. Therefore, a housekeeping index was defined with the geometric mean of the most stably expressed housekeeping candidates. These were *pmpA* and *groEL* for genes shared between *C*. *psittaci* and *C*. *abortus* and *hrtA* for genes shared among all three organisms. Gene expression was quantified using the 2^-(ΔΔCt)^ method. The log2-fold changes in gene expression determined by RNA-Seq and RT-qPCR were tested for linear association using the Pearson correlation coefficient.

### Species-specific and homologous genes

For the identification of species-specific and homologous genes, an all-vs.-all comparison of *C*. *psittaci*, *C*. *abortus* and *W*. *chondrophila* genomes was performed using RAST [61]. Identified bidirectional hits were filtered, and hits with less than 30% total DNA sequence identity were removed. In case of multiple matches only the best was retained. The best reciprocal hits by these criteria were considered as homologs. For the interspecies comparison of differentially expressed genes, the significance threshold *p*_*adj*_ < 0.01 and an absolute log2-fold change > 1.0 were applied.

### Biological functions and enrichment analysis

Biological functions were assigned to chlamydial genes using the RAST [61] database. To extend the list of categories, predictions for Incs, OMPs and Pmps were assembled from the literature [5, 19, 62]. In *C*. *psittaci* and *C. abortus*, potential T3SS effectors were predicted using EffectiveT3 [63], with a cutoff > 0.9999, and compared with the previously published SVM learning approach [5]. Genes predicted to be type III secreted by both methods were considered T3SS effectors. For *W*. *chondrophila*, only EffectiveT3 (cutoff > 0.9999) predictions were available. Differentially expressed genes in EBs or RBs within each subcategory were tested for over-representation using the one-sided Fisher’s exact test implemented in R version 3.1.0. Categories with less than two records were omitted in the Figures.

## Additional files

Additional file 1: Contains supplementary figures referred to in the text. Here, raw data processing, alignment and read distribution results are shown. Furthermore, the heatmaps of differentially expressed genes in *C. psittaci* and *C. abortus* are presented in higher resolution and NCBI locus tags, gene names and the functional annotations are included. Finally, the file contains the confirmation of the RNA-seq results with RT-qPCR for *dnaK*, *hctA*, *mip* and *omcB* shared among *C. psittaci*, *C. abortus* and *W. chondrophila*. (PDF 3185 kb)

Additional file 2: **Table S1.** Summary of alignment results and genome coverages. (XLS 9 kb)

Additional file 3: **Table S2.** Gene expression in *C. psittaci* EBs and RBs. (XLS 285 kb)

Additional file 4: **Table S3.** Gene expression in *C. abortus* EBs and RBs. (XLS 261 kb)

Additional file 5: **Table S4.** Gene expression in *W. chondrophila* EBs and RBs. (XLS 479 kb)

Additional file 6: **Table S5.** The expression of species-specific genes of *C. psittaci*, *C. abortus* and W. *chondrophila* is shown with a subdivision in genes not shared with any other known species. (XLS 38 kb)

Additional file 7: Species-specific genes involved in virulence. The expression of species-specific genes involved in virulence of *C. psittaci*, *C. abortus* and *W. chondrophila* is discussed. (DOC 23 kb)

Additional file 8: ** Table S6.** Expression of 354 homologous genes shared between *C. psittaci* and *C. abortus*. (XLS 173 kb)

Additional file 9: ** Table S7.** Expression of 562 homologous genes shared between *C. psittaci*, *C. abortus* and *W. Chondrophila*. (XLS 515 kb)

Additional file 10: ** Table S8.** Summary of primers used in the study. (XLS 11 kb)

## Abbreviations

cDNA: complementary DNA
EB: elementary body
hpi: hours post-inoculation
Inc: inclusion membrane protein
MOI: multiplicity of infection
OMC: outer membrane complex
OMP: outer membrane protein
Pmp: polymorphic membrane protein
PZ: plasticity zone
RB: reticulated body
RNA-Seq: RNA-sequencing
rRNA: ribosomal RNA
RT-qPCR: real-time quantitative polymerase chain reaction
SPG: sucrose-phosphate-glutamic acid
T3SS: type three secretion system
TARP: translocated actin recruiting phosphoprotein
tRNA: transfer RNA
