# Supplementary material for: Virulence-related comparative transcriptomics of infectious and non-infectious chlamydial particles
Source: BMC Genomics. 2018 Aug 2;19:575. doi: 10.1186/s12864-018-4961-x (PMC6090853; doi:10.1186/s12864-018-4961-x)
Supplement: Supplementary file 1 — Contains supplementary figures referred to in the text. Here, raw data processing, alignment and read distribution results are shown. Furthermore, the heatmaps of differentially expressed genes in C. psittaci and C. abortus are presented in higher resolution and NCBI locus tags, gene names and the functional annotations are included. Finally, the file contains the confirmation of the RNA-seq results with RT-qPCR for dnaK, hctA, mip and omcB shared among C. psittaci, C. abortus and W. chondrophila. (PDF 3185 kb) [file 12864_2018_4961_MOESM1_ESM.pdf]

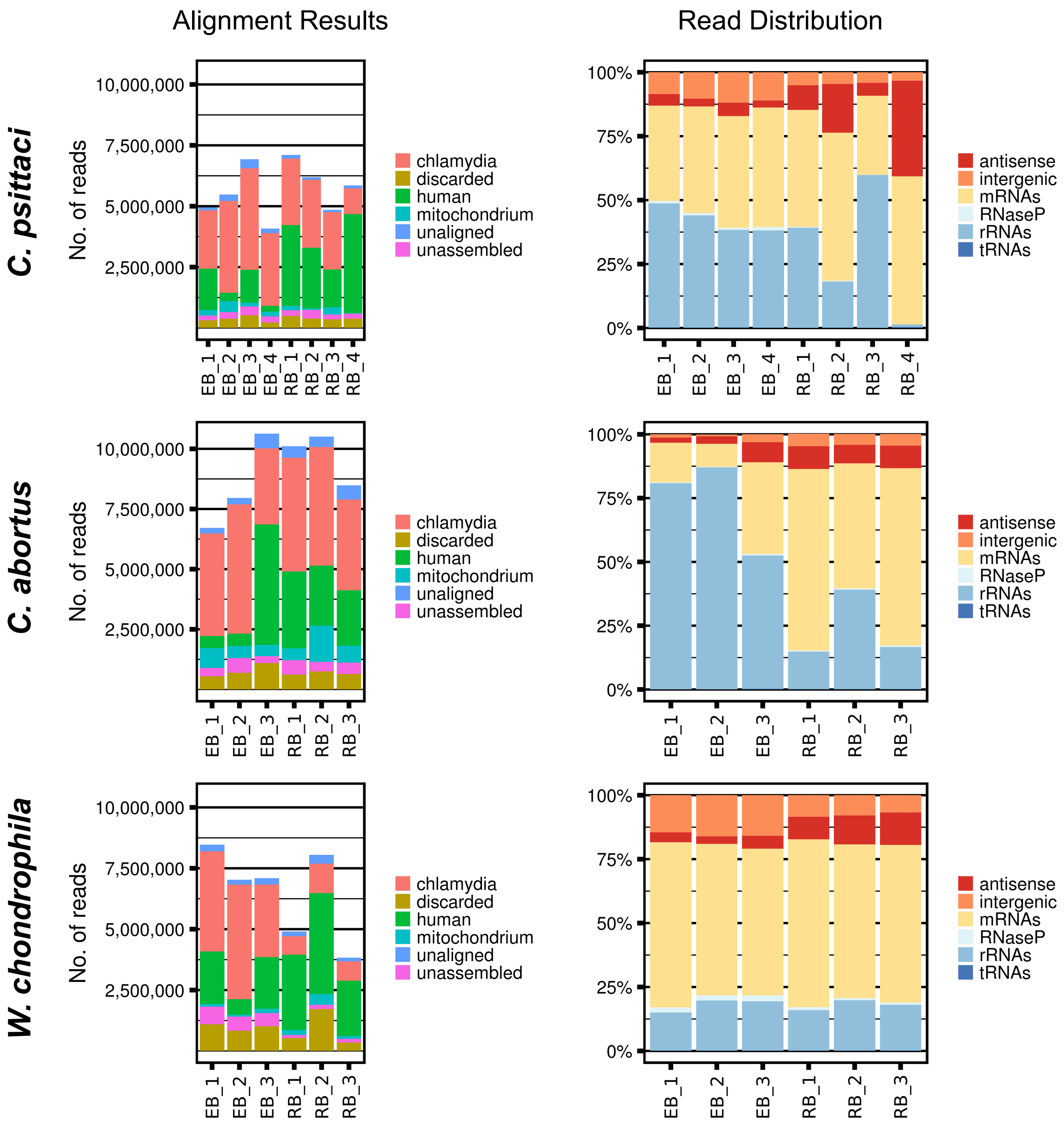

Figure S1. Sequencing reads of each library of *C. psittaci 02DC15*, *C. abortus 26/3* and *W. chondrophila 2032/99* were quality filtered, assembled and aligned to host and pathogen genomes (left panel). Reads that mapped to mitochondrial and human genome resemble host derived contaminations. However, most of the reads could be aligned to the chlamydial genomes and these were analyzed for distribution to various RNA classes (right panel).

Raw data processing and alignment results

A summary of the trimming, assembling and alignment results is shown in Figure S1 (left panel). Trimming of low quality and technical sequences resulted in 6% - 21% read depletion. The *W. chondrophila* libraries had a slightly lower quality than *C. psittaci* and *C. abortus* libraries. Because of overall short length of cDNA libraries the majority of reads were overlapping and assembled. In each sample a portion of reads mapped to the mitochondrial and human genome resembling minor host contaminations in EB and RB fractions. Non-coding RNAs, that make up to 95% of the total RNA content in a bacterial cell, were drastically reduced by rRNA depletion (Figure S1, right panel). Antisense and intergenic transcripts were detected in a range from 3.3% to 40.7% depending on the ratio of initially depleted rRNAs. Transcription in intergenic regions and antisense to known features in chlamydia was already demonstrated for major human pathogens *C. trachomatis* [1] and *C. pneumoniae* [2].

1. Albrecht M, Sharma CM, Reinhardt R, Vogel J, Rudel T. Deep sequencing-based discovery of the Chlamydia trachomatis transcriptome. Nucleic Acids Res. 2009;38(3): 868–77.
2. Albrecht M, Sharma CM, Dittrich MT, Müller T, Reinhardt R, Vogel J, et al. The transcriptional landscape of Chlamydia pneumoniae. Genome Biol. 2011;12(10): R98.

C. psittaci EB > *C. abortus* EB

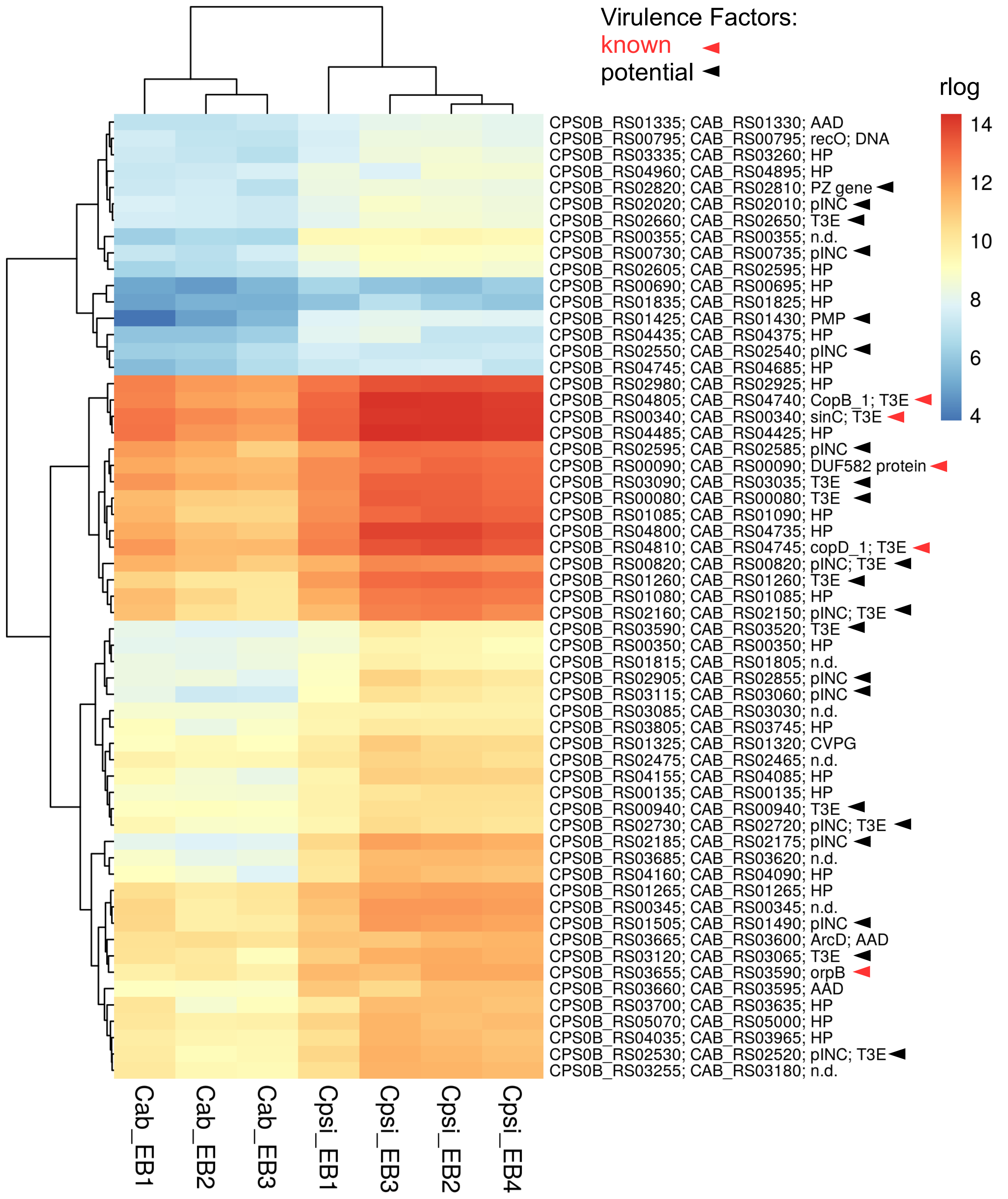

Figure S2. Heatmap showing the expression of 59 transcripts that are more abundant in EBs of *C. psittaci* than in *C. abortus*. Hierarchical clustering was performed using hclust with regularized log transformed (rlog) gene expression values of and the complete linkage method. The genes are specified by NCBI locus tags, gene names and the functional categories in which they are involved. Assigned functional categories are abbreviated as follows: Amino Acids and Derivatives (**AAD**); Cofactors, Vitamins and Prosthetic Groups (**CVPG**); DNA Metabolism (**DNA**); Gene within the Plasticity Zone (**PZ**); Hypothetical Protein (**HP**); not determined (**n.d.**); predicted Inclusion Membrane Protein (**pINC**); predicted T3SS Effector (**T3E**) and Polymorphic Membrane Protein (**PMP**).

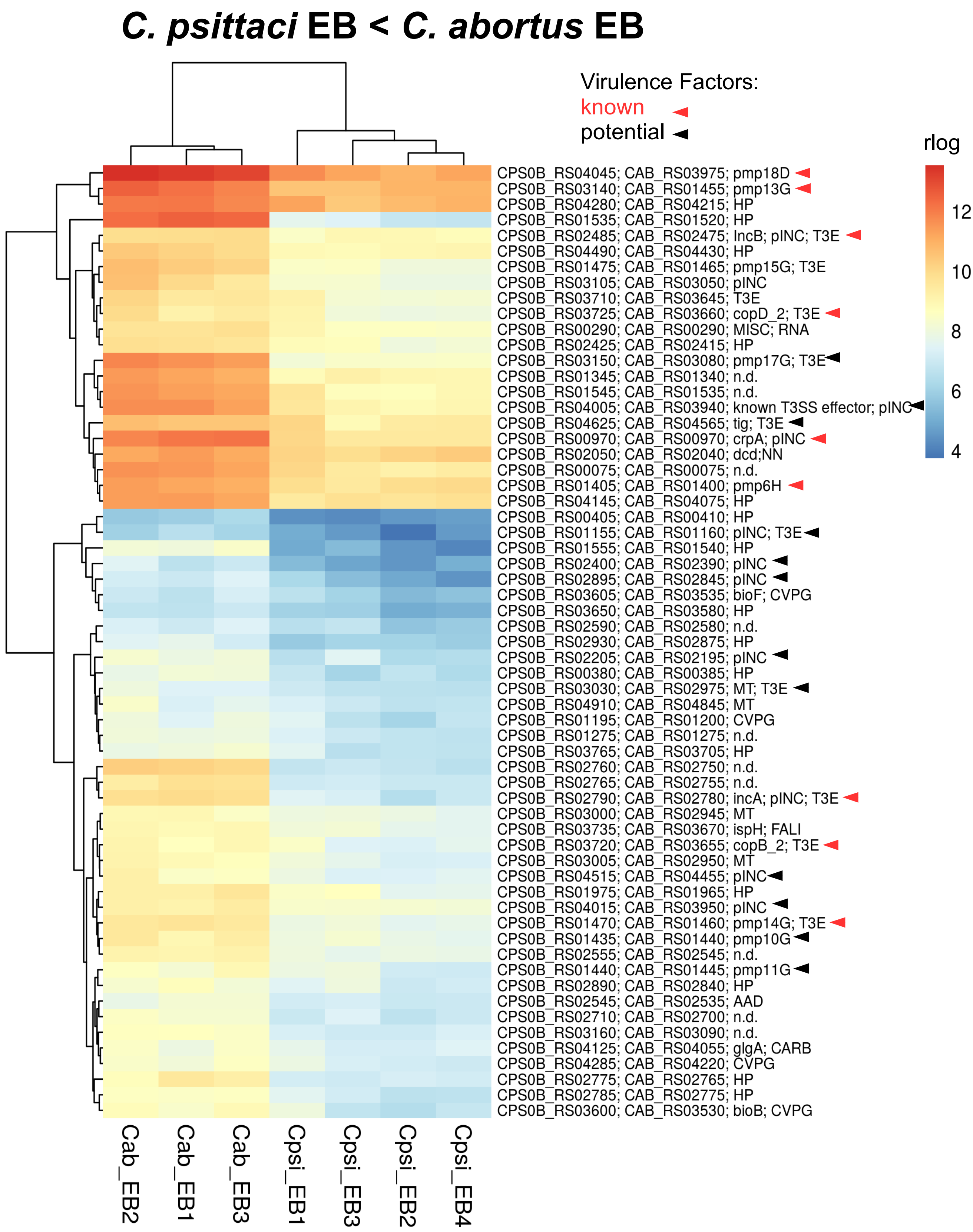

Figure S3. Heatmap showing the expression of 61 transcripts that are more abundant in EBs of *C. abortus* than in *C. psittaci*. Hierarchical clustering was performed using hclust with regularized log transformed (rlog) gene expression values of and the complete linkage method. The genes are specified by NCBI locus tags, gene names and the functional categories in which they are involved. Assigned functional categories are abbreviated as follows: Amino Acids and Derivatives (**AAD**); Carbohydrates (**CARB**); Cofactors, Vitamins and Prosthetic Groups (**CVPG**); Fatty Acids, Lipids and Isoprenoids (**FALI**); Hypothetical Protein (**HP**); Miscellaneous (**MISC**); Membrane Transport (**MT**); not determined (**n.d.**); Nucleosides and Nucleotides (**NN**); predicted Inclusion Membrane Protein (**pINC**); predicted T3SS Effector (**T3E**) and RNA Metabolism (**RNA**).

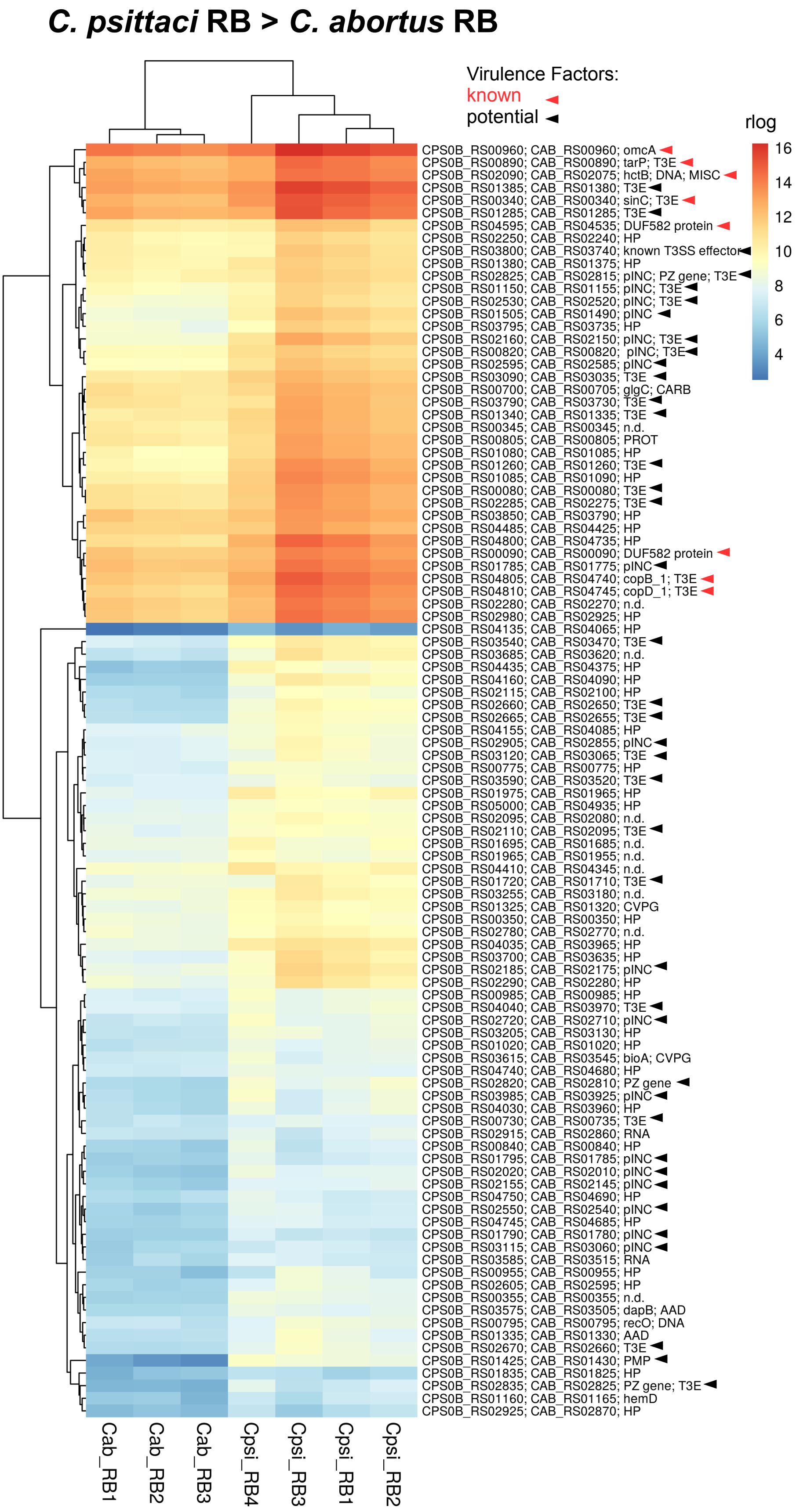

Figure S4. Heatmap showing the expression of 101 transcripts that are more abundant in RBs of *C. psittaci* than in *C. abortus*. Hierarchical clustering was performed using hclust with regularized log transformed (rlog) gene expression values of and the complete linkage method. Assigned functional categories are abbreviated as follows: Amino Acids and Derivatives (**AAD**); Carbohydrates (**CARB**); Cofactors, Vitamins and Prosthetic Groups (**CVPG**); DNA Metabolism (**DNA**); Gene within the Plasticity Zone (**PZ**); Hypothetical Protein (**HP**); not determined (**n.d.**); Miscellaneous (**MISC**); predicted Inclusion Membrane Protein (**pINC**); predicted T3SS Effector (**T3E**) and Polymorphic Membrane Protein (**PMP**); Protein Metabolism (**PROT**) and RNA Metabolism (RNA).

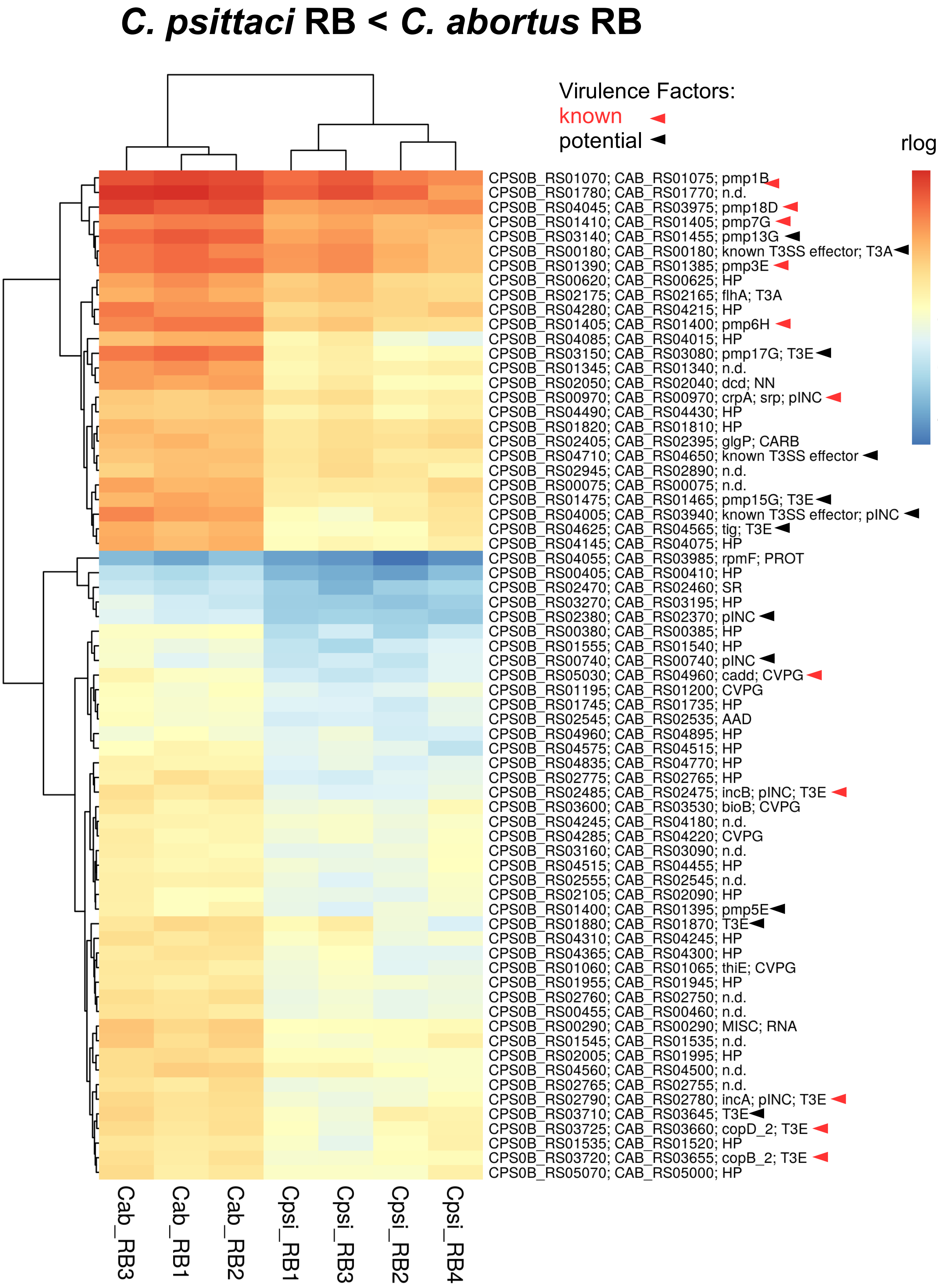

Figure S5. Heatmap showing the expression of 69 transcripts that are more abundant in RBs of *C. abortus* than in *C. psittaci*. Hierarchical clustering was performed using hclust with regularized log transformed (rlog) gene expression values of and the complete linkage method. The genes are specified by NCBI locus tags, gene names and the functional categories in which they are involved. Assigned functional categories are abbreviated as follows: Amino Acids and Derivatives (**AAD**); Carbohydrates (**CARB**); Cofactors, Vitamins and Prosthetic Groups (**CVPG**); Hypothetical Protein (**HP**); not determined (**n.d.**); Nucleosides and Nucleotides (**NN**); predicted Inclusion Membrane Protein (**pINC**); predicted T3SS Effector (**T3E**); Protein Metabolism (**PROT**); RNA Metabolism (**RNA**); Stress Response (**SR**) and T3SS Apparatus (**T3A**).

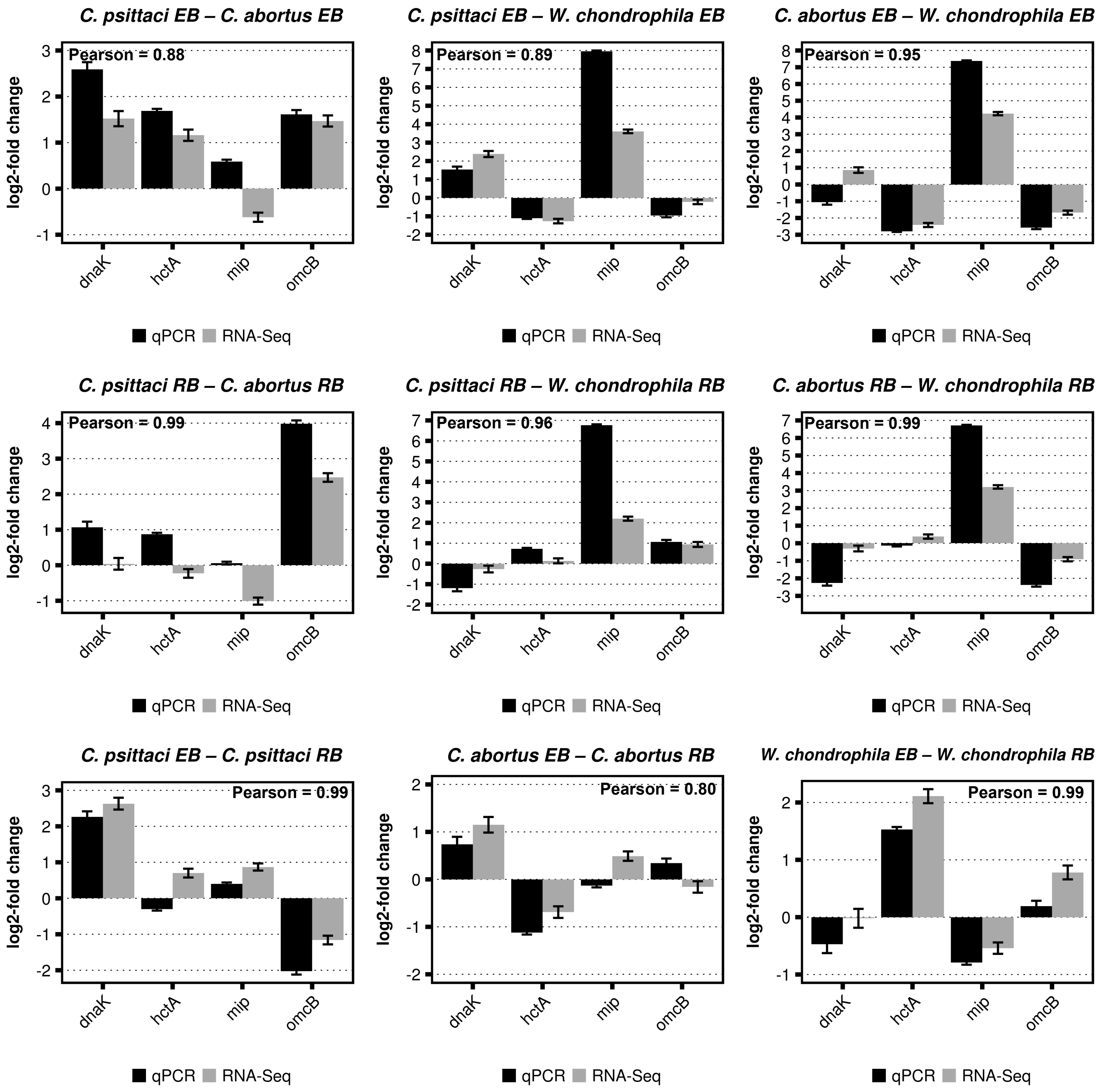

Figure S6. Confirmation of the RNA-seq results with RT-qPCR for four virulence factors shared among *C. psittaci*, *C. abortus* and *W. chondrophila*. Correlation between the log2 ratio of expression determined by RT-qPCR and RNA-seq in the inter-species comparison of EBs (upper panel) and RBs (mid-panel) as well as in the intra-species comparison (lower panel) are shown. Standard deviation of log2-fold changes are indicated. The Pearson correlation coefficients demonstrate a high degree of correlation between the RT-qPCR and RNA-seq results.
