# Supplementary material for: Virulence-related comparative transcriptomics of infectious and non-infectious chlamydial particles
Source: BMC Genomics. 2018 Aug 2;19:575. doi: 10.1186/s12864-018-4961-x (PMC6090853; doi:10.1186/s12864-018-4961-x)
Supplement: Supplementary file 7 — Species-specific genes involved in virulence. The expression of species-specific genes involved in virulence of C. psittaci, C. abortus and W. chondrophila is discussed. (DOC 23 kb) [file 12864_2018_4961_MOESM7_ESM.doc]

**Species-specific protein-coding genes**

***C. psittaci*-specific transcripts**

Of the 54 genes, which *C. psittaci* does not share with *C. abortus* or *W. chondrophila* (Figure 1A**)**, 25 encode Pmps, predicted Incs or T3SS effectors and are putatively involved in virulence (Table S5). The remaining 29 genes encode 24 hypothetical proteins or are involved in biosynthetic processes. A known virulence factor present is the MACPF domain gene which is similarly expressed in EBs and RBs. The chlamydia MACPF domain proteins are putatively involved in host immune avoidance [1] or have a pore-forming function [2,3].

A potential virulence factor similarly expressed in EBs and RBs is the Inc CPS0B_RS01350. In addition, the *C. psittaci* genome encodes six Pmps and seven PZ genes. Most of the Pmps are similarly expressed but CPS0B_RS01445 and CPS0B_RS01455 are up-regulated in RBs (Table S5). Two genes within the PZ i.e. CPS0B_RS02845 and CPS0B_RS02850 are predicted to be T3SS effectors and are highly up-regulated in EBs. In addition, the *C. psittaci* genome encodes 8 hypothetical proteins that have no homologs in any other known organism.

***C. abortus-*specific transcripts**

The expression of 16 *C. abortus* genes, which are not shared with *C. psittaci* and *W. chondrophila* are shown in (Table S5). Annotated as putative virulence factors are the highly expressed Inc (CAB_RS03935) and a Pmp (CAB_RS01425). The remaining genes encode the adenylate kinase CAB_RS03700 and 13 hypothetical proteins. For six of these no homologs were found in any other organism. Two of the hypothetical proteins i.e. CAB_RS01525 and CAB_RS01705 might be involved in infection because they are highly up-regulated in EBs.

***W. chondrophila*-specific transcripts**

In total, *W. chondrophila* contains 1271 protein-coding genes which are not shared with *C. psittaci* or *C. abortus* (Figure 1A). Among these are 31 hypothetical proteins with no homologs in any other organism (Table S5). Known *W. chondrophila* specific virulence factors participate in stress response, drug resistance or belong to the *W. chondrophila* ompA family (Table S5). Involved in stress response are for example the superoxide dismutase WCW_RS05925, the catalase WCW_RS03140 and the nitric oxide reductase WCW_RS02305. The enzymes detoxify reactive oxygen species (ROS) and nitric oxide (NO), are produced by the host as an early response upon chlamydial infection [4]⁠. Consistent is that the expression of these enzymes is up-regulated in EBs, which indicates ROS and NO detoxification in the early infection. Other protective mechanisms such as pH-homeostasis are based on numerous sodium and potassium transporters as well as on the carbonic anhydrase WCW_RS00730, which import protons into the bacterium [5]. Most components of these transporters are similarly expressed in EBs and RBs (Table S5). Some genes like WCW_RS08890 and WCW_RS0506 are predicted T3SS effectors and up-regulated in EBs and might therefore be involved in infection. Other putative virulence factors are the 11 novel outer membrane proteins (Omps) recently found in *W. chondrophila*. These encode putative porins, which might facilitate *W. chondrophila* infection but the detailed functions are unknown [6]⁠. The majority of the Omps are differentially expressed in EBs and RBs.

**References**

1. Stebbins CE, Galán JE. Structural mimicry in bacterial virulence. Nature. 2001;412(6848): 701–5.

2. Ishino T, Chinzei Y, Yuda M. A Plasmodium sporozoite protein with a membrane attack complex domain is required for breaching the liver sinusoidal cell layer prior to hepatocyte infection. Cell Microbiol. 2005;7(2): 199–208.

3. Kafsack BFC, Pena JDO, Coppens I, Ravindran S, Boothroyd JC, Carruthers VB. Rapid membrane disruption by a perforin-like protein facilitates parasite exit from host cells. Science. 2009;323(5913): 530–3.

4. Azenabor AA, Mahony JB. Generation of reactive oxygen species and formation and membrane lipid peroxides in cells infected with Chlamydia trachomatis. Int J Infect Dis. 2000;4(1): 46–50.

5. Bertelli C, Collyn F, Croxatto A, Rückert C, Polkinghorne A, Kebbi-Beghdadi C, et al. The waddlia genome: A window into chlamydial biology. PLoS One. 2010;5(5): e10890.

6. Bertelli C, Collyn F, Croxatto A, Rückert C, Polkinghorne A, Kebbi-Beghdadi C, et al. The waddlia genome: A window into chlamydial biology. PLoS One. 2010;5(5): e10890.
